# Neuronal silence as a predictive biomarker and target for epileptic seizures suppression

**DOI:** 10.1038/s41598-026-44063-w

**Published:** 2026-04-09

**Authors:** Diogo L. M. Souza, Lucas E. Bentivoglio, Enrique C. Gabrick, Paulo R. Protachevicz, Iberê L. Caldas, Kelly C. Iarosz, Salvador Dura-Bernal, Antonio M. Batista, Fernando S. Borges

**Affiliations:** 1 Graduate Program in Sciences, State University of Ponta Grossa, Ponta Grossa, Paraná Brazil; 2 Institute of Physics, University of São Paulo, São Paulo, Brazil; 3 Graduate Program in Electrical Engineering and Industrial Informatics, Federal University of Technology of Paraná, Curitiba, Paraná Brazil; 4 Department of Physiology and Pharmacology, State University of New York Downstate Health Sciences University, New York, NY USA; 5 Center for Biomedical Imaging and Neuromodulation, The Nathan S. Kline Institute for Psychiatric Research, New York, USA; 6 Department of Mathematics and Statistics, State University of Ponta Grossa, Ponta Grossa, Paraná Brazil; 7 Center for Mathematics, Computation, and Cognition, Federal University of ABC, São Bernardo do Campo, São Paulo Brazil

**Keywords:** Epilepsy, Seizure prediction, Seizure Suppression, Intermittent synchronization, Biomarkers, Neurology, Neuroscience

## Abstract

Epilepsy is a prevalent neurological disorder marked by abnormal synchronized neuronal firing, which can often lead to long-term cognitive and physical impairments. In this work, we introduce a reliable biomarker for seizure prediction. Through simulations of a conductance-based neuronal network model that reproduces spontaneous seizure-like events, we identify that slow potassium channels play an important role in seizure generation. Our key finding is the consistent presence of a prolonged period of neuronal silence that precedes the seizure onset, establishing it as a physiologically relevant biomarker for seizure prediction. Notably, this silence is also identified in human electrophysiological data, confirming its physiological and clinical relevance. Based on this biomarker, we develop a targeted suppression strategy that, in our simulations, significantly shortens long seizure duration by up to 93%. Our results establish the network silence as a predictive and clinically translatable biomarker for seizure dynamics, opening new avenues for improved forecasting and personalized neuromodulation therapies in epilepsy.

## Introduction

Epilepsy is a worldwide concern affecting over 52 million people^1^. This neurological disorder evokes spontaneous or induced seizures, which may lead to cognitive, psychological, and physical consequences^1–3^. The International League Against Epilepsy (ILAE) characterizes seizures as a transient abnormal neuronal activity or excessive synchronization in the brain^4–6^. Several studies have been conducted in order to deepen our knowledge regarding the mechanisms underlying seizures^7–13^. Protachevicz et al.^14^ showed that a reduction in inhibition within a neuronal network facilitates synchronization. The authors demonstrated that for some coupling the abnormal synchronization emerges in the network in a hysteresis loop, where the network can display asynchronous and synchronous states depending on the initial state. Lopes et al.^7^ studied how the structure and the neuronal tissue heterogeneity influence the emergence of different types of seizures. The authors reported that the interplay between both properties greatly affects which seizure types the network can display. Studies suggest that the generation of abnormal synchronization is a result of the imbalance between excitation and inhibition^8,14^.

The electrochemical activity of neurons is mediated by ions that enter or leave cells according to their transmembrane electrochemical gradient. This ionic current is generated by the opening of a membrane channel for a specific ion and is responsible for synaptic transmission and the generation of the action potential. Therefore, its dynamics can also contribute to the maintenance and termination of abnormal activity during epileptic seizures^15^. For instance, the dynamics of K$^+$ channels can be associated with several levels with epileptic seizures. They contribute directly to the control of the neuronal excitability and also to the initiation of seizures^16^. During seizures, the K$^+$ extracellular concentration increases over baseline, which then depolarizes the cell, facilitating the production of bursts^15^.

Throughout the years, researchers have shown that epilepsy is characterized as a dynamical brain disorder^17–22^. The activity of epileptic rats was shown to be characterized as intermittent dynamics^20,23–25^. In this dynamics, periods of regular activity are irregularly alternated with chaotic activity without external inputs^26^. For an epileptic brain, the intermittent activity is characterized by the transition from asynchronous spikes (down state) to abnormal synchronous bursts (up states) and vice versa. Velazquez et al.^19^ experimentally showed that humans with partial epilepsy demonstrate intermittent dynamics during seizures.

The unpredictability of seizures is one of the main factors that severely impacts the lives of individuals with epilepsy. Despite all theoretical and experimental advances in neuroscience, the trigger mechanisms of seizures are not yet well understood^18^. Predicting abnormal synchronization remains a critical challenge, and its resolution can improve the lives of those affected by this brain disorder. Therefore, further studies in trigger mechanisms and prediction can increase our understanding of abnormal synchronization phenomena and provide new possible approaches for treating epileptic seizures. Seizure prediction and forecasting are two distinct approaches in epilepsy research, each with its own methodology and objectives. Seizure prediction involves identifying specific preictal periods and issuing alarms when a seizure is expected to occur within a defined timeframe. In contrast, seizure forecasting assesses the probability of a seizure occurring over a longer horizon, providing a continuous risk assessment rather than a definitive prediction^27^. Both approaches aim to anticipate seizure events and differ in their methods and applications. Forecasting, prediction, and triggers of abnormal synchronization in the brain have been an area of intense research interest in neuroscience^28–33^. Maturana et al.^29^ reported that iEEG signals of epilepsy patients display some characteristics of critical slowing down, which could serve as a biomarker for predictive algorithms. Batista et al.^30^ demonstrated that a cumulative EEG-based metric can improve seizure prediction compared to standard approaches.

One approach that has been extensively used in neuroscience is computational modeling^34–36^ due to the possibility of exploring new hypotheses and understanding the role of multiple processes that underlie neuronal activity. In this work, we construct a biophysical neuronal network model that mimics spontaneous epileptic seizures^8^. Meanwhile we include the slow potassium channel dynamics^37^ to better approximate our simulations to real human neurons^16^. Biophysical, or conductance-based, models have been used to investigate both healthy and pathological neuronal activities due to their ability to represent complex channel dynamics^38,39^. First, we developed an epileptic model of a random network capable of reproducing spontaneous seizures based on synaptic weight. Next, we analyze the phenomena that precede the onset of abnormal synchronization states. To validate our findings, we employ a supervised machine learning algorithm to predict the emergence of abnormal synchronization based on the novel biomarker that we introduce. Finally, we validated our approach using experimental data from epilepsy patients (data available in^40^) and expand our analysis to a biologically reconstructed human temporal cortex network used as a representation of a spontaneous seizure-generating network^41^.

Our findings reveal that, immediately before a seizure onset, the neuronal network exhibits a prolonged period of silence. This phenomenon contrasts with previous observations of brief silence periods in auditory cortex recordings during neuronal spike correlations^42^. Neuronal firing reductions have been reported at seizure onset when the iEEG shows low-voltage fast activity (LVFA) during presurgical intracranial monitoring in patients with focal, drug-resistant epilepsies^40,43^. Consistent with this, several animal models suggest that focal seizures can begin with a transient increase in GABAergic activity that suppresses principal-cell firing while promoting elevations in extracellular K+, which in turn increase network excitability, facilitate recruitment of surrounding neurons, and support seizure progression^9^. We introduce a novel metric “mean silence time” to detect and predict the emergence of abnormal synchronization. We demonstrate that the transition from healthy asynchronous spiking to pathological synchronization occurs when the mean silence time surpasses a critical threshold. This extended silence leads to a reduction in the slow potassium current, reducing neuronal adaptability and facilitating burst generation. Furthermore, we implement a DBS-like suppression strategy, inspired by Deep Brain Stimulation^44–46^, employing targeted stimulation on a select group of neurons, upon seizure prediction, which remarkably shortens the duration of abnormal synchronization in approximately 93%. Showing that our prediction method across different network architectures. Our results establish the mean silence time as a reliable biomarker for seizure prediction and highlight the crucial role of the slow potassium current in seizure generation. Most importantly, our work holds the potential to refine clinical treatment strategies by utilizing the predictive capability of neuronal silence.

## Methods

To ensure the generality of our findings, we first constructed a random network composed of $N = 1000$ neurons, with $80\%$ excitatory and $20\%$ inhibitory cells^47^. The connectivity followed an Erdös–Rényi topology with a connection probability of $p = 0.1$, excluding self-connections^8^. This initial model served as a simplified framework to explore the fundamental mechanisms underlying the observed dynamics.

To validate the generality of these results, we subsequently employed a biologically detailed network reconstructed from the human temporal cortex^41^. This dataset, made available by Shapson-Coe et al.^41^, represents a petavoxel-scale volume of human cerebral cortex imaged and reconstructed at nanometer resolution using serial-section electron microscopy. It provides comprehensive connectivity data among tens of thousands of neurons, capturing the intricate microcircuit architecture and synaptic organization of the temporal cortex with unprecedented accuracy. We subsampled only the neurons located within 100 $\mu$m of the dataset center, thereby minimizing artifacts introduced by severed connections near the volume boundaries. In this subsampled network, $71\%$ of the neurons are excitatory and $29\%$ are inhibitory. The sampled volume spans multiple cortical layers (L1 to L6), allowing for a heterogeneous distribution of excitatory and inhibitory neurons consistent with the organization of the human temporal cortex. Each neuron retains the connectivity profile defined in the original reconstruction.

We extended our theoretical findings from the network models to experimental data recorded and published by Elahian et al.^40^, which investigated low-voltage fast seizures in human patients. The dataset comprises multiunit recordings from the left anterior hippocampus (LAH) and right entorhinal cortex (REC) obtained using intracranial depth electrodes in patients with pharmacoresistant epilepsy. The datasets analyzed in this study were originally recorded and made publicly available by Elahian et al.^40^ in the Zenodo repository^48^.

In both biologically detailed and random network configurations, the membrane potential ($V_i$) of each neuron was governed by a conductance-based model described by

1 $$\begin{aligned} C\frac{dV_i}{dt} = -g_{\textrm{leak}}(V_i - E_{\textrm{leak}}) - I^i_{\textrm{K}} + I^i_{\textrm{Na}} + I^i_{\textrm{M}} + I_{\textrm{ext}} + I^i_{\textrm{sup}} + \sum _{j = 1}^{N}(V_j^{\textrm{rev}} - V_i) g_j M_{ij}, \end{aligned}$$

where *C* is the membrane capacitance, $g_{\textrm{leak}}$ the leak conductance, and $E_{\textrm{leak}}$ the resting potential. $I_{\textrm{ext}}$ is a constant external current applied to all neurons, and $I^i_{\textrm{sup}}$ represents the suppression current applied individually to neuron *i*. The last term accounts for chemical synapses received by neuron *i*, where the reversal potential $V_j^{\textrm{rev}}$ depends on whether neuron *j* is excitatory ($V_j^{\textrm{rev}} = 0$ mV) or inhibitory ($V_j^{\textrm{rev}} = -80$ mV). The model parameters were set to $C = 1~\mu \mathrm{F/cm^2}$, $g_{\textrm{leak}} = 0.01~\mathrm{mS/cm^2}$, $E_{\textrm{leak}} = -85~\textrm{mV}$, and $I_{\textrm{ext}} = 175~\textrm{pA}$.

Synaptic conductances evolve according to $dg_i/dt = -g_i / \tau _s$. When neuron *i* fires ($V_i> 0$ mV), its conductance is updated as $g_i \rightarrow g_i + g_{\textrm{syn}}$^8,49^, where $g_{\textrm{syn}}$ denotes the coupling strength. Excitatory and inhibitory synapses were defined as $g_{\textrm{exc}} = g_{\textrm{syn}}$ and $g_{\textrm{inh}} = 4g_{\textrm{syn}}$, respectively, ensuring balanced network excitability. The adjacency matrix $M_{ij}$ encodes the connectivity pattern between neurons. The ionic currents $I^i_{\textrm{K}}$ and $I^i_{\textrm{Na}}$ follow the original Hodgkin–Huxley formalism^50^, while $I_{\textrm{M}}$ represents a slow potassium current. Further details of the ion channels model are provided in the Supplementary Information.

To measure the synchronization degree of the network, we consider the Kuramoto order parameter^49,51^ defined by

2 $$\begin{aligned} \textrm{R}(t) = \frac{1}{N} \left| \sum _{j=1}^N \exp {(i \varphi _j(t))} \right| , \end{aligned}$$

where $\varphi _j(t) = 2m\pi + 2m \frac{t - t_j^m}{t_j^{m+1} - t_j^{m}}$ is the phase of a neuron *i*, *m* is the *m*-th firing of the *j*-neuron and $t_j^m$ is the time of the *m*-th action potential generated. The interval of *t* is defined in $t^{m}_j< t < t^{m+1}_j$ and it is updated when $t>t^{m+1}_j$. The mean value of R throughout the time is given by $\overline{\textrm{R}} = \frac{1}{\Delta t} \int _{t_{\textrm{initial}}}^{t_{\textrm{final}}} \textrm{R}(t)$, where $\Delta t=t_{\textrm{final}}-t_{\textrm{initial}}$ is the time window of analysis. The values of R(*t*) and $\overline{\textrm{R}}$ range from 0 to 1, where 1 indicates a total synchronization across the network, 0 represents that the neurons are desynchronized and values between 0 and 1 mean that the network is partially synchronized.

Neurons exhibit a wide range of complex activities, for instance spikes and bursts. To detect spikes or burst activity in the whole network, we calculate the coefficient of variation (CV)^49^ given by $\overline{\textrm{CV}} = \frac{\sigma _{\textrm{ISI}}}{\overline{\textrm{ISI}}}$, where $\sigma _{\textrm{ISI}}$ is the standard deviation of the ISIs ($\textrm{ISI}_i^m= t_i^{m+1}-t_i^m$) and $\overline{\textrm{ISI}}$ is the mean inter spike interval of the network ($\overline{\textrm{ISI}} = \frac{1}{N} \sum ^N_{i=1} \frac{1}{M_i} \sum ^{M_i}_{m=1} \textrm{ISI}_i^m$, where $M_i$ is the number of ISIs)^52^. The spike activity produces $\overline{\textrm{CV}}<0.5$, whereas the burst behavior generates a $\overline{\textrm{CV}} \ge 0.5$ due to the ISIs high variability along bursts. In our simulations, we confirm that adopting a threshold value of $\textrm{CV}=0.5$ is sufficient to reliably distinguish spiking from bursting activity, thereby providing a clear criterion for spike-burst classification, in agreement with previous studies^5,8,14,49^. In spontaneous seizures, the spike and burst activities alternate intermittently over time. Therefore, we also compute an instantaneous coefficient of variation CV(*t*). For each time *t*, 7 ISIs before and 7 ISIs after the time *t* were used in the computation. We consider a total of 14 ISIs for each time point, which provides a sufficient sample to reliably capture the instantaneous dynamics of the network. Using a very small number of ISIs, such as two or three, ca lead to an inaccurate characterization, whereas incorporating an excessively large number of ISIs substantially increases the computational cost without a corresponding improvement in our results.

We define the silence duration $T_i$ of a neuron *i* as $T_i(t) = t - t^i_{m},$ where $t^i_m$ is the instant of time at which the *i*-th neuron fires. The interval of *t* is defined as $t^i_{m+1}<t<t^i_m$, *m* updates every time $t>t^i_{m+1}$. To evaluate the silence duration of the whole network, we compute the mean silence duration of the network

3 $$\begin{aligned} \langle \textrm{T}\rangle =\frac{1}{N}\sum _{i=1}^{N}T_i(t). \end{aligned}$$

## Results

In this section, we present our results of the random neuronal network, experimental data, and human cortical network.

### Random neuronal network

**Fig. 1:**
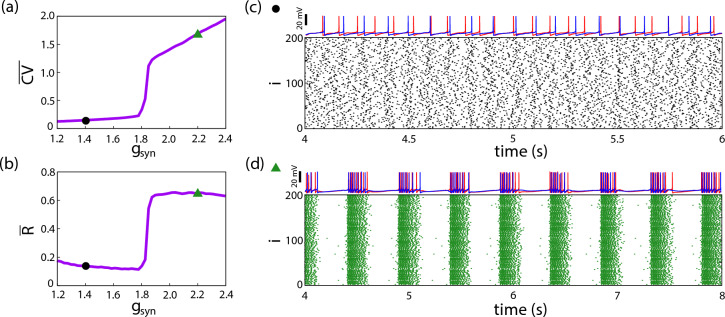
The effect of coupling strength ($g_{\textrm{syn}}$) on network dynamics. (**a**) Average coefficient of variation ($\overline{\textrm{CV}}$), where black circles indicate asynchronous spiking and green triangles indicate synchronized bursting. (**b**) Mean Kuramoto order parameter ($\overline{R}$). (**c**) Raster plot of asynchronous activity at $g_{\textrm{syn}} = 1.4$ $\mu \mathrm{S/cm^2}$ (black circle). (**d**) Raster plot of synchronized bursting at $g_{\textrm{syn}} = 2.2$ $\mu \mathrm{S/cm^2}$ (green triangle).

An increase in excitatory synapses or a reduction in inhibition can lead to abnormal burst synchronization in neuronal networks^8^. Figure 1 shows (a) the mean coefficient of variation ($\overline{\textrm{CV}}$) and (b) the mean Kuramoto order parameter ($\overline{\textrm{R}}$) as functions of the coupling strength $g_{\textrm{syn}}$. A transition from asynchronous spiking to synchronous bursting occurs for $g_{\textrm{syn}}>1.8\, \mu \mathrm{S/cm^2}$. Panels (c) and (d) display raster plots, where each dot marks the firing of a neuron *i*. For $g_{\textrm{syn}} = 1.4\, \mu \mathrm{S/cm^2}$ (panel (c), black circle), the neurons exhibit desynchronized spiking, characteristic of physiological activity. In contrast, panel (d) (green triangle) shows that for $g_{\textrm{syn}} = 2.2\, \mu \mathrm{S/cm^2}$, the neurons exhibit abnormal burst synchronization, a trait of epileptic seizures.

For $g_{\textrm{syn}}$ values near the transition from spike desynchronization to burst synchronization (Figures 1(a) and 1(b)), intermittent states of abnormal synchronization are observed. In these states, both spike desynchronization (down state) and burst synchronization (up state) alternate over time. During down state $\textrm{CV}(t)<0.5$ and $\textrm{R}(t)<0.6$, which characterizes spike desynchronization, whereas in up states CV$(t)\ge 0.5$ and $\textrm{R}(t) \ge 0.6$ indicating burst synchronization. The up states are associated with self-initiated and self-terminated seizures^5,19,26^. In this work, we first investigate these intermittent dynamics using synchronization metrics and then propose a novel method to predict up states.

**Fig. 2:**
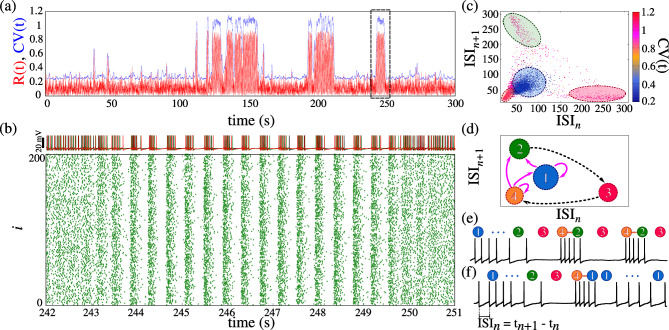
Intermittent network activity. (**a**) The instantaneous coefficient of variation, CV(*t*) (blue), and R(*t*) (red) are shown for a synaptic conductance of $g_{\textrm{syn}} = 1.825\ \mu \mathrm{S/cm^2}$. (**b**) The membrane potentials of three neurons and the corresponding raster plot are displayed for the interval highlighted by the dashed rectangle in (a). (**c**) The $\textrm{ISI}_n$ versus $\textrm{ISI}_{n+1}$ distribution is presented, with the color bar indicating the instantaneous CV. (**d**) A schematic of the clusters identified in (c) is provided, dashed arrows show cluster transitions, while magenta solid arrows indicate potential cluster transitions. (**e**) and (**f**) display examplar voltage trace in the spike-to-burst and burst-to-spike cluster transitions, respectively. The large circles denote clusters and the smaller colored circles indicate repeated occurrences until a transition occurs.

An intermittent state in our model is displayed in Figure 2. Panel (a) shows the Kuramoto order parameter (red line) and the instantaneous CV(t) (blue line) for $g_{\textrm{syn}}=1.825\, \mu \mathrm{S/cm^2}$. Although these metrics are computed independently, they exhibit similar patterns during intermittent states. The duration of both down and up states varies throughout the time series. As shown in Figure 2(a), a few up states are relatively short (varying from 3 s to 20 s), while others last longer. Panel (b) displays the raster plot and the voltage trace of three neurons of the time interval highlighted by the black dashed rectangle in Figure 1(a). Initially, during this interval, neurons fire at random times until a burst synchronization emerges. The up state self-terminates after a period of time, and the network returns to the down state^53^.

Neuronal interspike-intervals (ISI$_n=t_{n+1}-t_n$) in the intermittent state are irregular due to the alternation between asynchronous spikes and synchronous burst activity. Panel (c) displays the $(\textrm{ISI}_{n+1}, \textrm{ISI}_n)$ distribution for 30% of the network neurons during the highlighted interval, with each point’s color depicting the instantaneous CV at the $n$th spike. This distribution can be examined from two perspectives: a broad view that captures the overall up-to-down state transition and a detailed view focusing on specific activity transitions. In the broad analysis, two clusters emerge: blue for down states (CV$(t) < 0.5$) and pinkish for up states (CV$(t)\ge 0.5$). The zoomed analysis reveals four distinct clusters by considering the CV(t) values and the temporal distance among the clusters. The blue and orange clusters are difficult to distinguish based on their temporal separation; however, their CV(t) and ISI values exhibit different dynamics. Very short ISIs ([10, 40] ms) correspond to interburst activity, whereas medium-lasting ISIs ([50, 90] ms) are associated with regular spiking. The transitions among these clusters are schematized in panel (d), where the network begins in cluster 1 (blue, sustained down state), remains there until a prolonged silence gives rise to a shift into cluster 2 (green, indicating a transition to longer interburst ISIs). Cluster 2 represents the move from short to long ISI durations (the interburst interval), followed by a transition to cluster 3 (pink, pre-burst ISIs) immediately before burst onset. Once burst activity commences, the network enters cluster 4 (orange, active burst) and then either returns to cluster 2 (producing another burst) or reverts to cluster 1 (terminating the burst cycle). Panels (e) and (f) display these dynamic cluster transitions over time, with numbered circles above the voltage trace indicating the current cluster. Notably, immediately before the transition from cluster 1 to 2, a down-to-up state transition, the majority of neurons exhibit a global “silence”.

**Fig. 3:**
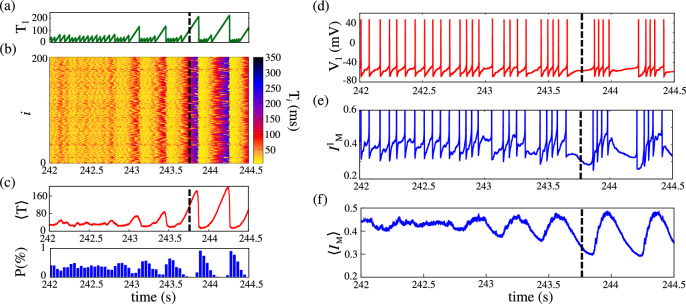
Neuronal silence and slow potassium dynamics preceding an up state. (**a**) Silence duration $T_1(t)$ over time for a representative neuron, showing extended silence before the up state onset. (**b**) Time evolution of silence duration across all neurons, color indicates activity level, bright for active firing, dark red for prolonged silence. (**c**) Mean network silence duration $\langle T \rangle$ (top) and population spike histogram (bottom). (**d**) Membrane potential $V_i(t)$ of a single neuron. (**e**) Slow potassium current $I^i_{\textrm{M}}$ of the same neuron. (**f**) Mean slow potassium current $\langle I_{\textrm{M}} \rangle$ across the network. The vertical dashed line in all panels marks the onset of the up state. We consider $g_{\textrm{syn}} = 1.825\, \mu \mathrm{S/cm^2}$.

Immediately before an up state, neurons exhibit a period of silence. To quantify this behavior, we calculate the silence duration $T_i(t)$ for each neuron and examine the dynamics of the slow potassium current; channel that plays a critical role in burst generation and seizure activity^16,54^. Figure 3 summarizes these findings. Panel (a) shows the silence duration $T_1$ over time for a representative neuron of the network ($i=1$), revealing a prolonged silent period just before the onset of an up state. Panel (b) displays the silence duration across all neurons, where the color bar indicates silence duration, bright colors denote active firing, while red/dark colors indicate prolonged silence. In panel (c), the mean silence duration $\langle T \rangle$ is shown along with the population spike histogram below it. Panels (d) and (e) depict, respectively, the membrane potential and the slow potassium current $I_{\textrm{M}}$ of a single neuron, while panel (f) shows the network-averaged $\langle I_{\textrm{M}} \rangle$. The dashed line in all panels marks the onset of an up state. Before this transition, neurons fire asynchronously with silence durations under 90 ms. As the network approaches the up state, most neurons exhibit silence periods longer than 120 ms, which then triggers burst activity. Panel (c) confirms that during the down state, $\langle T \rangle$ remains close to zero. Although it rises sharply before synchronization onset (as marked by the dashed line). Panels (d) and (e) reveal that while the voltage and $I^i_{\textrm{M}}$ follow similar dynamics, $I^i_{\textrm{M}}$ is notably lower during the transition. Panel (f) further shows that prolonged silence reduces $\langle I_{\textrm{M}} \rangle$.

The main novelty of this work is the introduction of $\langle T \rangle$, which can be measured directly in real neurons, through appropriate techniques. We evidence that $\langle T \rangle$ can be employed as a predictor feature of synchronization, i.e., *R*. To do that, we employ the Random Forest algorithm, which is a supervised machine learning method composed of multiple decision trees^55^. Initially, the model splits the given data into $M=200$ random subsets, then each subset passes through a decision tree. The predicted Kuramoto order parameter ($\textrm{R}_{\textrm{pred}}$) is determined by averaging the predictions from all trees. We consider the GridSearchCV package from the sklearn library to choose the best hyperparameters for the model. Our input data consists of two lagged values of the mean silence time, each one with a duration of 10 ms. Thus, to predict $\textrm{R}_{\textrm{pred}}$ at time *t*, the algorithm uses the delayed values $\langle \textrm{T} \rangle (t - \textrm{lag})$ and $\langle \textrm{T} \rangle (t - 2\textrm{lag})$. This approach quantifies how $\langle T \rangle$ may regulate network synchrony ($\textrm{R}_{\textrm{pred}}$). More details about the Random Forest algorithm can be found in the Supplementary Information file, Supplementary Fig. S1 displays a schematic of the Random Forest algorithm.

**Fig. 4:**
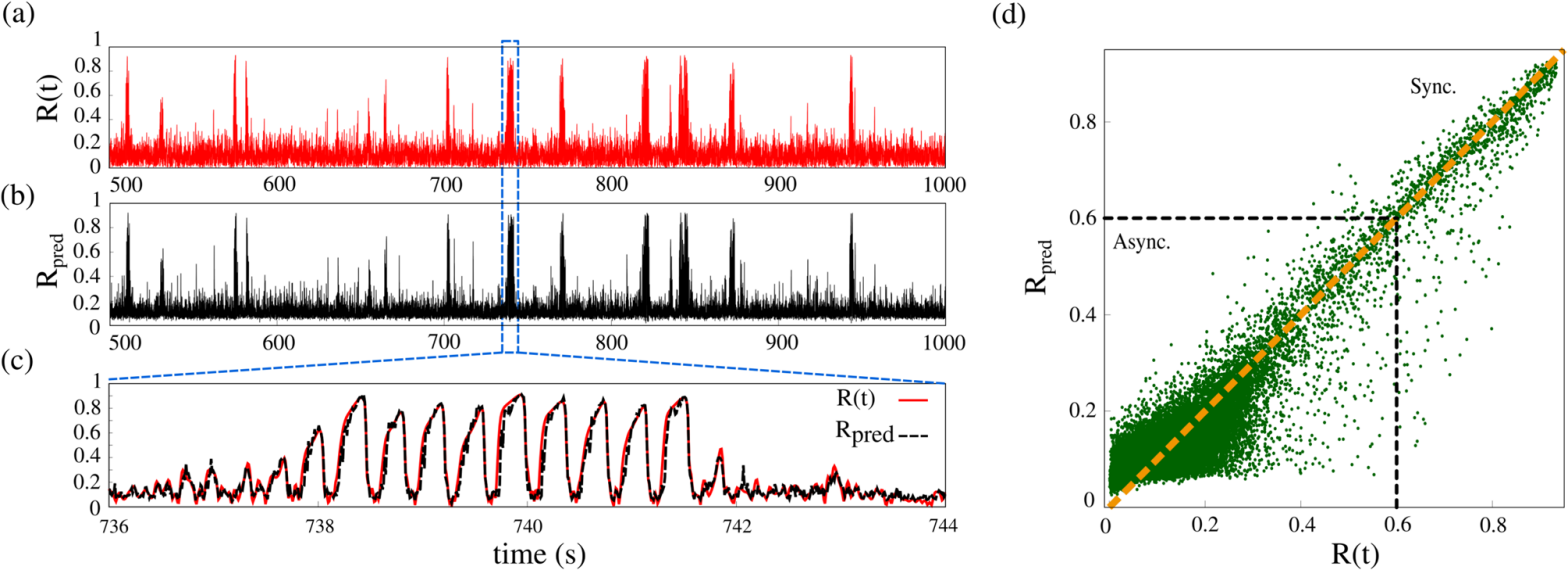
(**a**) Network simulated Kuramoto order parameter $\textrm{R}(t)$ and (**b**) the predicted Kuramoto order parameter $\textrm{R}_{\textrm{pred}}$ using 50 % of $\langle T \rangle$ time-series to train the Random Forest algorithm. (**c**) A zoom-in of the blue rectangle in (**a**) and (**b**). (**d**) Distribution of $\textrm{R}(t) \times \textrm{R}_{\textrm{pred}}$, where the dashed line indicates the ideal prediction ($\textrm{R}_{\textrm{pred}} = \textrm{R}(t)$). Table 1 shows the performance of the Random Forest for different $g_{\textrm{syn}}$ values.

Figure 4 shows (a) *R*(*t*) obtained from the simulation of the model, (b) the predicted Kuramoto order parameter $R_{\textrm{pred}}$, and (c) their comparison during the highlighted up state. The algorithm is trained with 50$\%$ of the original $\langle T \rangle$ time series, $g_{\textrm{syn}}=1.811$ nS, and then used to predict $\textrm{R}_{\textrm{pred}}$ for the remaining duration. The algorithm effectively captures the transitions between down and up states based solely on $\langle T \rangle$, evidencing the predictive power of this quantity and its importance in practical applications. Panel (c) zooms into an up state (blue dashed rectangle), where the transition from asynchronous to synchronous activity is clearly observed. Panel (d) shows a parity plot comparing $\textrm{R}_{\textrm{pred}}$ and R(*t*). The yellow line marks a perfect prediction ($\textrm{R}_{\textrm{pred}}=\textrm{R}(t)$), while the black dashed lines separate asynchronous and synchronous regimes. In asynchronous states, data points are scattered due to the high variability of R(*t*) in this state. The value of $\textrm{R}_{\textrm{pred}}$ indicates the presence of down states. In contrast, during synchronous activity, points cluster near the yellow line, demonstrating accurate prediction of up states. These results indicate that network silence time $\langle T \rangle$ can reliably predict both down and up states and their transitions. The high accuracy in detecting up states highlights its potential as a tool for early seizure prediction.

We evaluate the performance of the Random Forest algorithm in predicting $\textrm{R}(t)$ based on $\langle T \rangle$ for different synaptic conductance values. These couplings were chosen for their significant impact on up states, including seizure-like event occurrence and duration. Table 1 exhibits $R^2$, Pearson correlation coefficient (*r*), and the mean absolute error (MAE) for various $g_{\textrm{syn}}$ values. The model is trained with a single dataset, 50% of the $\langle T \rangle$ time series of the simulation with $g_{\textrm{syn}} = 1.811\, \mu \mathrm{S/cm^2}$. For all considered $g_{\textrm{syn}}$ values, the MAE value remains below 0.1, indicating that the algorithm generalizes well in predicting the transitions between up and down states. The error increases with higher synaptic conductance, due to elevated firing rates and prolonged up states of higher couplings. This result underscores the potential of mean silence time as a biomarker for predicting spontaneous seizures. Supplementary Fig. S2 displays the $\textrm{R}(t)$ and $\mathrm{R_{pred}}$ time series for different $g_{\textrm{syn}}$ values in Table 1.

**Table 1:** Performance of the Random Forest algorithm across different synaptic conductance values ($g_{\textrm{syn}}$). The evaluation includes the coefficient of determination ($R^2$), Pearson correlation coefficient (*r*), and mean absolute error (MAE).

$g_{\textrm{syn}} (\mu \mathrm{S/cm}^2)$	$R^2$	*r*	Mean Absolute Error
1.811	0.864	0.930	0.031
1.825	0.876	0.937	0.047
1.830	0.884	0.942	0.051
1.835	0.885	0.946	0.063

**Fig. 5:**
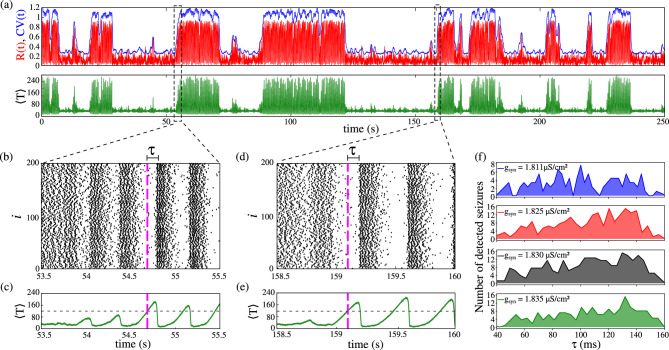
Intermittent network activity. (**a**) Instantaneous coefficient of variation (CV(*t*)), Kuramoto order parameter (R(*t*)), and mean silence time ($\langle T \rangle$) are shown over time. (**b**) Raster plot and (**c**) corresponding $\langle T \rangle$ for the interval highlighted by one of the dashed rectangles. (**d**) and (**e**) display the raster plot and $\langle T \rangle$ for the other highlighted time interval, respectively. The magenta dashed line indicates the predicted up-state onset, while the black dashed line marks the mean silence time threshold ($\langle T \rangle = 120$ ms). (**f**) Distribution of the predicted time ($\tau$) for different $g_{\textrm{syn}}$. Except panel (**f**), we consider $g_{\textrm{syn}} = 1.835\, \mu \mathrm{S/cm^2}$.

The $\langle T \rangle$ value is related to the emergence of up states. We develop a novel up state predictor based entirely on $\langle T \rangle$. Figure 5 displays an intermittent state for $g_{\textrm{syn}}=1.835\,\mu \mathrm{S/cm^2}$. Panel (a) shows the time series of $\textrm{R}(t)$ (red), CV(*t*) (blue), and $\langle T \rangle$ (green). During down states, $\textrm{R}(t)$ and CV(*t*) remain below 0.6 while $\langle T \rangle$ stays under 120 ms. In contrast, the network becomes increasingly silent before an up state emerges, with $\langle T \rangle$ only exceeding 120 ms at the down-to-up state transition. Dashed black rectangles in panel (a) highlight the down-to-up transitions. For example, panel (b) shows a raster plot from 53.5 s to 55.5 s, where asynchronous spiking coincides with low $\langle T \rangle$, which then increases just before the up state onset (panel (c)). A similar pattern is observed between 158.5 s and 160 s in panels (d) and (e). Additional raster plots of the time series during a few interictal periods and the onset are provided in Supplementary Fig. S3. Notably, if $\langle T \rangle$ increases without surpassing 120 ms, only asynchronous bursts occur, confirming that prolonged silence is crucial for the transition. We hypothesize that sustained silence ($\langle \textrm{T} \rangle \ge 120$ ms) allows slow K$^+$ currents to reach a critical minimum, thereby reducing neuronal adaptation and enabling synchronized bursts (Figures 3 (e) and (f) display a sudden reduction in the slow K$^+$ current). Defining the up state onset as the moment when $\langle \textrm{T} \rangle> 120$ ms (instant represented by the magenta dashed line in panels (b)–(e)), we denote the time difference between the predicted up state and the onset of the burst synchronization as $\tau$. In both raster plots, our prediction of an up state is a few milliseconds before burst synchronization, $\tau = 110$ ms in panel (b) and $\tau = 97$ ms in panel (d). Panel (f) presents the histogram of $\tau$ over a 5000 s simulation for distinct coupling strengths, demonstrating that most up states are predicted in $40 \le \tau \le 160$ ms before onset.

**Fig. 6:**
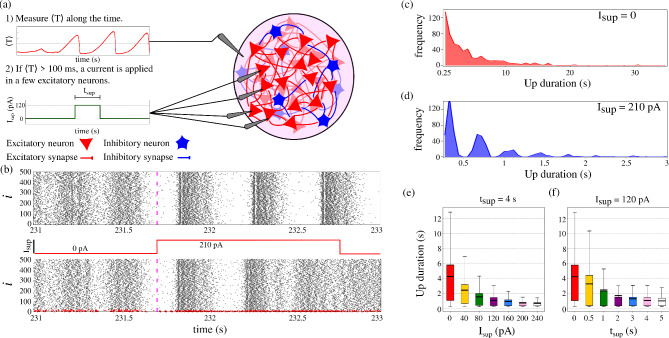
(**a**) Schematic of the suppression method. (**b**) Raster plots of an up state without suppression current (top panel) and with a suppression current of $I_{\textrm{sup}}=210$ pA (bottom panel). Red dots mark the neurons that are targeted by the suppression current and are displayed in red for the entire duration of the time series. (**c**) and (**d**) Histogram of up state durations for $I_{\textrm{sup}}=0$ and $I_{\textrm{sup}}=210$ pA, respectively. (**e**) Box plot of up-state durations for various $I_{\textrm{sup}}$ values, and (**f**) box plot for different suppression durations $t_{\textrm{sup}}$. The solid black line denotes the mean, and the dashed white line represents the median. We consider $g_{\textrm{syn}}=1.83\, \mu \mathrm{S/cm^2}$.

Since our method accurately predicts up states, we implement a suppression method based on deep brain stimulation (DBS)^44–46^. Deep brain stimulation is a method that consists of the chronic application of a stimulus in a region of the brain for treating different brain disorders, such as epilepsy^46^ and Parkinson’s disease^56^. DBS can be implemented either in an open-loop (continuous) or closed-loop (adaptive)^57,58^. In open-loop DBS, stimulation is delivered continuously and independently of the ongoing neural dynamics, whereas in closed-loop approaches the stimulation is modulated in real time based on measured neural activity. Closed-loop DBS has been proposed as a strategy to reduce unnecessary stimulation, minimize side effects, and improve energy efficiency. In this work, we adopt a closed-loop, adaptive DBS-inspired framework in which an excitatory stimulus is triggered only when an up state is predicted and is maintained for a predefined time interval. We consider an excitatory suppression current to perturb the silence of the network that precedes an up state. The excitatory stimulus likely reduces pre-seizure network silence ($\langle T \rangle$), preventing the slow K$^+$ current from reaching a low value and then stabilizing a down state. Figure 6(a) displays the schematic representation of the suppression method. The suppression methodology is described as follows: 1) we monitor the mean silence time of the network; 2) Once it surpasses 100 ms, an excitatory constant current is applied in 1% of the excitatory neurons for a fixed time duration $t_{\textrm{sup}}$ and then the current is turned off.

Panel (b) presents raster plots of an up state in the absence (top panel) and in the presence (bottom panel) of an excitatory suppression current ($I_{\textrm{sup}}=210$ pA). The magenta dashed line marks the instant at which the seizure is predicted, triggering the stimulation for a duration of $t_{\textrm{sup}}=4$ s at $g_{\textrm{syn}}=1.83\,\mu \mathrm {S/cm^2}$. Additional raster plots illustrating the effects of varying $I_{\textrm{sup}}$ and $t_{\textrm{sup}}$ are provided in Supplementary Figure S4. Prior to the prediction, $I_{\textrm{sup}}$ remains inactive. Once triggered, the current leads to a network transition to a down state approximately 1 s later. Panels (c) and (d) show histograms of up-state durations without suppression ($I_{\textrm{sup}}=0$) and with suppression current ($I_{\textrm{sup}}=210$ pA), respectively. The up states exceed 30 s when no suppression current is applied and it is reduced to under 4 s with suppression, a reduction of approximately 93%. Panels (e) and (f) exhibit box plots of up-state durations for different $I_{\textrm{sup}}$ intensities and suppression durations. The black solid line indicates the mean duration and the thin black bar represents the standard deviations from the mean. An increase in $I_{\textrm{sup}}$ narrows the variability and reduces the mean duration. However, there is no substantial reduction in the up-state duration for more intense currents ($I_{\textrm{sup}}>200$ pA). When comparing the up-state durations for $I_{\textrm{sup}}=200$ and $I_{\textrm{sup}}=240$ pA using the Mann-Whitney U test, we obtain a p-value of 0.0336, which is statistically significant. Nevertheless, the corresponding rank-biserial correlation is only 0.0727, an effect size considered negligible^59^. These underscores that statistical significance does not necessarily imply biological relevance^60^ and that increasing the stimulation amplitude does not substantially shorten up states. Moreover, $t_{\textrm{sup}}$ shorter than 0.5 s does not significantly impact up-state variability, while durations above 1 s considerably decrease seizure length. We observe that stimulating a higher proportion of cells (5%, 10% and 15%) can further reduce seizure duration compared with stimulating only 1%. For instance, the mean up-state duration decreases when increasing the stimulated fraction from 1% to 5%, dropping from approximately 1 s to about 0.35 s. Further increases to 10% and 15% lead only to minor additional reductions, with mean durations of 0.31 s and 0.3 s, respectively. Importantly, increasing the stimulated proportion also qualitatively alters the network dynamics, leading to a less stable regime characterized by more frequent seizure-like events. This effect is consistent with the fact that perturbing a larger fraction of neurons effectively increases the overall external current of the network, which can shift the overall dynamical state of the network and promote different patterns of activity. In contrast, perturbing only a small subset of neurons does not transition the system to a different dynamical regime, but rather modulates the ongoing activity within the same regime. For these reason, we focus on perturbing 1% of the neuronal population, which is sufficient to shorten seizure durations while remaining minimally invasive. Unlike continuous DBS, our approach limits exposure to external stimuli, offering a novel and targeted method for seizure suppression. These results demonstrate that the mean silence time is not only a reliable predictor of up states, it also enables an efficient suppression strategy.

### Experimental data

**Fig. 7:**
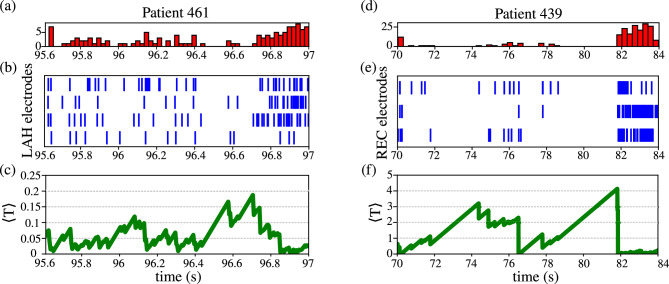
Longer silence observed preceding a seizure of two distinct patients (**a**), (**b**), and (**c**) are from patient 461 and (**d**), (**e**), and (**f**) from patient 439. (**a**) and (**d**) firing histogram. (**b**) and (**e**) raster plots of their respective electrodes. (**c**) and (**f**) mean silence time of the patient. The name of the electrodes represents their location: LAH = left anterior hippocampus and REC = right entorhinal cortex.

Since our simulation results on prediction and seizure suppression are accurate and efficient, we extend our analysis to experimental data from Elahian et al.^40^. The authors recorded local field potentials (LFPs) from epileptic patients using intracranial electrodes. For our analysis, we selected two patients (461 and 439) whose recordings contained a sufficient number of spikes to allow meaningful comparisons, and for whom the neuronal activity immediately preceding seizure onset was clearly visible. However, only a portion of the dataset described in^40^ has been made publicly available through^48^. The recording sites differed between patients: in patient 461, electrodes were implanted in the left anterior hippocampus (LAH), while in patient 439, they were placed in the right entorhinal cortex (REC). Figures 7(a–c) show the analysis for Patient 461 while Figures 7(d–f) for Patient 439. Figures 7(a) and 7(d) display the number of detected spikes over time for patients 461 and 439, respectively. Panels (b) and (e) depict raster plots of the intracranial electrodes, and panels (c) and (f) display the corresponding mean silence times. In patient 461, we observe desynchronization and low-frequency activity before seizure onset. During the down states, the number of spikes remains below 5 (panel (a)). As the seizure starts, the spiking activity increases, as depicted in panels (a) and (b). Notably, just before seizure onset, a prolonged period of neuronal silence occurs with mean silence time increasing in panel (c), precisely the signature that our model predicts. A similar pattern is observed in patient 439, as shown in panels (d), (e), and (f). Immediately before the seizure, the entorhinal cortex also exhibits a prolonged silence. The overall number of spikes is lower for this patient, which is expected given the generally lower activity of cortical regions compared to the hippocampus^61,62^. This concordance between experimental recordings and our simulations reinforces mean silence time as a reliable biomarker for seizure onset. It also confirms that the core mechanism preceding a seizure in our model is present in human epileptic networks.

### Human cortical network

**Fig. 8:**
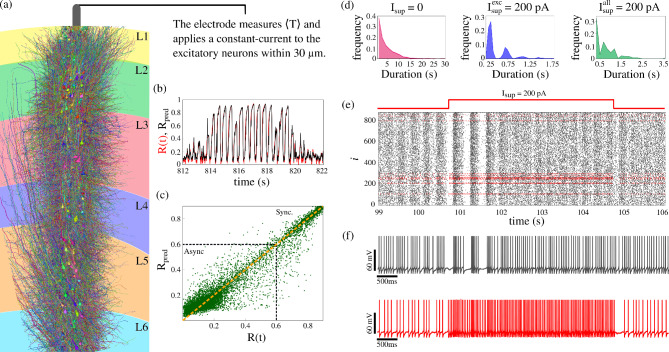
Human temporal cortex network simulation. (**a**) Representation of the temporal human cortical network^41^. (**b**) The Kuramoto order parameter *R*(*t*) (red line) and the Random Forest prediction $\textrm{R}_{\textrm{pred}}$. (**c**) Distribution of $\textrm{R}(t) \times \textrm{R}_{\textrm{pred}}$. This Random Forest algorithm produces a mean absolute error$=$0.058, $r=0.951$, and $R^2=0.903$. (**d**) Histogram of the up-state duration for $I_{\textrm{sup}}=0$ (pinkish line) and $I_{\textrm{sup}}=200$ pA. The blue curve corresponds to the case in which only excitatory neurons receive the stimulus ($I_{\textrm{sup}}^{\textrm{exc}}$), whereas the green curve represents the condition in which both excitatory and inhibitory neurons ($I_{\textrm{sup}}^{\textrm{all}}$) are subjected to $I_{\textrm{sup}}=200$ pA. (**e**) Raster plot of the network activity during the suppression of an up state, where the red dots indicate neurons receiving the suppression current. (**f**) The voltage trace of a neuron that does not receive the suppression current (gray line) and one that receives the suppression current (red line).

Due to the fact that an abstract random network can not fully capture the complexity of human brain connectivity, we also analyze a network reconstructed from the human temporal cortex^41^. To further validate our findings with regard to the role of neuronal silence in seizure prediction, we apply our method to this biologically based network and verify the presence of spontaneous seizures. Figure 8(a) illustrates the network considered. Similar to the random network, this biological network also exhibits up states, as shown in panel (b), which displays the Kuramoto order parameter $\textrm{R}(t)$ (red line) alongside the Random Forest prediction $\textrm{R}_{\textrm{pred}}$ (blue line) during a transition from a down to an up state. The corresponding network activity during the down-to-up state transition is shown in Supplementary Video V1. The Random Forest model is trained using the approach described in the previous section, where the input consists of the mean silence time $\langle T \rangle$ with two time delays of 10 ms. As with the random network, the model accurately distinguishes between the desynchronized healthy state and the abnormally synchronized pathological state. Panel (c) shows the distribution of $\textrm{R}(t)$ versus $\textrm{R}_{\textrm{pred}}$. Although some dispersion is observed in the asynchronous regime, the model still correctly identifies the ongoing network state. During the up states, the predicted values approximately align with the ideal prediction line, indicating the algorithm’s predictive performance. These results further support the mean silence time as a biologically meaningful feature for anticipating the transitions from healthy states to pathological activities.

To test whether the suppression strategy previously applied to the random network also works in a biologically based model, we apply the same protocol to the human cortical network. Specifically, an excitatory current is applied to excitatory neurons within 30 $\mu$m of the electrode position ($x=1100$ $\mu$m, $y=2000$ $\mu$m) whenever an up state is predicted. The stimulation lasts for 4 seconds before being turned off. In panel (d), we computed histograms of the up-state durations for $I_{\textrm{sup}}=0$ (pinkish line) and $I_{\textrm{sup}}=200$ pA. When only excitatory neurons received the stimulus, the results are shown in blue, whereas when both excitatory and inhibitory neurons are subjected to $I_{\textrm{sup}}=200$ pA, they are shown in green. Applying the stimulus exclusively to excitatory neurons shortens the up-state duration more effectively than stimulating both neuron types. We note, however, that the “excitatory only” scenario is a theoretical simplification of DBS. The application of the suppression current reduces the seizure duration by approximately 88% when both excitatory and inhibitory neurons are stimulated and 93% when only excitatory neurons are stimulated, highlighting the effectiveness of the mean silence time as a predictive biomarker for seizure intervention. The raster plot in panel (e) displays the initiation of an up state that is suppressed after the current application. The red dots indicate neurons receiving the stimulation. A few milliseconds after the stimulation start, a brief burst of activity is observed, which is then rapidly suppressed. Panel (f) shows the voltage traces of a neuron not receiving the suppression current (gray line) and one that does (red line). The non-stimulated neuron exhibits a short burst of activity before suppression, whereas the stimulated neuron does not enter an up state. As expected, during the current application, the stimulated neuron fires at a higher frequency and returns to baseline after the stimulation end. A video illustrating both scenarios, with and without suppression current, is provided in Supplementary video V2. These findings confirm that the mean silence time is a biomarker for seizure prediction and an effective trigger for timely suppression of pathological activity in a human cortical network. This supports its potential use in clinical interventions on neurological disorders.

## Discussion and conclusions

As a key contribution, we introduce a biomarker based on the duration of neuronal silence preceding up states, which we refer to as mean silence time. We observe that longer periods of neuronal silence reliably precede the onset of up states, providing a predictive window for seizure onset. Leveraging this feature, we develop a deep brain stimulation strategy that shortens up-state duration by approximately 93%, demonstrating its therapeutic potential. Furthermore, we validate our findings using experimental data from human epileptic patients, confirming that prolonged neuronal silence is a reliable pre-seizure marker. To establish these results, we present a conductance-based neuronal network model that reproduces abnormal synchronization patterns resembling spontaneous epileptic seizures. We first analyze a random network and then extend our investigation to a biologically based human temporal cortex network. In both cases, the models alternate irregularly between a healthy asynchronous “down state” and a burst-synchronized “up state” that mimics seizure-like activity. To capture the critical role of neuronal adaptation, we incorporate slow potassium (K$^+$) channels into the model, consistent with previous findings that deficiencies in these channels can facilitate burst generation^15,16^. Together, these results advance our mechanistic understanding of seizure dynamics and open new avenues for improving seizure forecasting and clinical intervention in epilepsy.

Before the onset of an up state, the slow K$^+$ current decreases to low levels, reducing neuronal adaptability and facilitating burst generation across most neurons in the network. We propose that the duration of neuronal silence, the interval during which neurons remain inactive, plays a pivotal role in this process. Notably, Mochol et al.^42^ reported brief, stochastic transitions into population-level silence in auditory cortex that underlie noise correlations, suggesting that population silence is a general network phenomenon. Our prolonged pre-seizure silence may represent the same mechanism operating on longer timescales in epileptic networks. Our findings indicate that the overall network activity reduces prior to an abnormal synchronization, leading to a decrease in slow K$^+$ current. The decrease in slow K$^+$ current is directly related to the appearance of bursts in pyramidal cells. We recently showed that this channel is fundamental in the synchronization of bursts^8^. Additionally, intracellular recordings showed that blocking slow K$^+$ current by linopirdine induces intrinsic bursting in pyramidal cells^63^. Moreover, recordings of a seizure-­like event (SLE) in whole guinea pig brain preparation and biophysically realistic computational model also showed that seizure initiation is related to low excitatory spiking and the initial accumulation of extracellular K$^+$ due to intense interneuronal spiking^9^. Within this physiological context, feedforward or surround inhibition, known to oppose ictal propagation, has also been proposed as a mechanism contributing to transient reductions in firing in tissue adjacent to a seizure core^64–67^. Rather than being mutually exclusive, inhibitory restraint and extracellular K$^+$-driven excitability can act at different spatial or temporal scales, jointly shaping the transition toward pathological synchronization. Although the mean silence duration fluctuates over time, it consistently increases as the network nears an up state, ultimately surpassing a critical threshold of 120 ms. This finding supports the potential of silence duration as a biomarker for predicting the onset of pathological synchronization.

Independent of the firing frequency, the networks consistently enter a silent phase immediately before an up state, establishing silence as a biomarker for seizure detection and prediction. To validate this correlation, we employed a Random Forest algorithm trained on silence duration to predict the Kuramoto order parameter. The algorithm reliably predicts the down-to-up state transition, reinforcing the close relationship between neuronal silence and synchronization. Based on this result, we develop a mean silence duration-based predictor that identifies abnormal synchronization approximately [40, 160] ms before its onset, clearly linking network silence to the emergence of seizure-like states. Importantly, this prolonged silence is not only a computational feature, it also emerges consistently in experimental data from human epileptic patients. We observed this behavior in two distinct brain regions, the hippocampus and the cortex, suggesting that neuronal silence is a pre-seizure marker across different regions. Multiple mechanisms contribute to spontaneous epileptic seizures in humans. Our method is designed to predict seizure onset at the network level. Accordingly, in this work we restrict our analysis to the modeled network and perturbations applied to neurons within this network. Investigating propagation mechanisms, including feedforward inhibition, would require extending the model to multiple interacting cortical columns. In such an extended framework, one column would generate seizures while neighboring columns would be used to examine propagation-related effects^10^ such as excitatory-neuron quiescence. In the present work, however, we consider a single column only.

Other proposed seizure-prediction biomarkers emphasize different features of the brain’s activity prior to seizure onset. For example, time-varying trends in high-frequency oscillation rates have been shown to distinguish periods preceding seizures from normal baseline activity^68^. Likewise, theoretical analyses treat seizure onset as a critical phase transition, predicting that neural signals will exhibit “critical slowing down”, manifested as increased variance and autocorrelation, as a seizure approaches^29^. These approaches focus on ramping activity or connectivity changes, whereas our silence-duration metric instead captures the converse phenomenon: a sustained quiescent period immediately before the rapid emergence of network synchrony. Importantly, prolonged pre-seizure silence has begun to appear in experimental studies as well. In a rodent model of epileptic spasms, neocortical neurons entered a striking “pause” of firing just before seizure onset, a pause that shares many features with the down-states of sleep slow waves^69^. We hypothesize that this emerging pre-up-state silence may be mechanistically linked to the decrease in neuronal synchronization reported in EEG recordings prior to seizure onset^70,71^. In this framework, the reduction in firing activity during the silent period may contribute to an effective desynchronization of the network, consistent with observations of reduced synchrony in the pre-ictal state^70^. As the seizure develops, these temporarily quiescent neurons may then be rapidly recruited into the pathological, highly synchronized up state. Notably, the occurrence and magnitude of pre-seizure quiescence can vary across brain regions, seizure types, and experimental conditions. Together, these convergent findings suggest that neuronal silence is a marker of impending seizure activity, reinforcing our computational results and highlighting silence as a predictive biomarker alongside other dynamical measures.

Intracranial human recordings demonstrated that low voltage fast activity (LVFA) is the most common EEG-onset pattern observed in focal seizures/epilepsies^13,43^. Others seizure-onset patterns as low-frequency high-amplitude periodic spikes, sharp activity, spike-and-wave activity, burst of high-amplitude polyspikes, burst suppression, and delta brush are less common and may have different biomarker as high frequency oscillation (HFO)^13^. Additionally, we included data from two patients solely to illustrate how this quiescent period can be quantified from human iEEG recordings. Consistent with this observation, other presurgical intracranial studies using microelectrode arrays in patients with focal epilepsy have reported reduced neuronal firing at seizure onset^11^. In contrast, recordings focusing on the early ictal period in surrounding regions indicate comparatively lower and less entrained activity relative to the ictal core, while firing rates remain above baseline^64^.

We implemented a closed-loop DBS-like protocol delivering brief excitatory pulses to a subset of neurons whenever prolonged network silence indicated an up-state. In both our random and human cortical models, this intervention reduced up-state durations by 93%. This suggests that prolonged silence serves as a reliable abnormal synchronization marker and aligns with efforts to use state-dependent stimulation to disrupt seizures. For instance, Ge et al.^72^ modeled seizure suppression in an absence-epilepsy model as a feedback control problem and showed that a neural-network controller could steer thalamocortical dynamics back toward normal oscillations. In vivo closed-loop neuromodulation has likewise proven effective: Ferrero et al.^73^ reported that hippocampal stimulation timed to interictal spikes prevents seizure spread and Dong et al.^74^ showed that midline thalamic DBS can suppress generalized seizures while enhancing vigilance. Beyond seizure suppression, a key advantage of the proposed approach is its local and brief nature, which restricts stimulation to a small subset of neurons within the epileptic focus. At the same time, neuronal silence also occurs during physiological states such as NREM sleep^75,76^ and a suppression strategy based solely on silence detection could interfere with such states if applied beyond the focus. Addressing this limitation will require the integration of additional biomarkers and state-dependent control mechanisms.

We theorize that the self-termination of up states can occur through a mechanism opposite to that underlying the silence preceding seizure onset. Based on our observations, increasing the activity of a small subset of neurons, seizure onset appears to be sufficient to shorten seizure duration. Within this interpretation, enhanced spiking can shift the self-termination process closer to the onset of the up state. Furthermore, self-termination, both in the presence and absence of a suppression current, may be associated with sustained or elevated neuronal activity that prevents the adaptation current from decaying to low values. This, in turn, is able to inhibit fast spiking and hinder the prolonged maintenance of the up state. Together, these converging results illustrate that precisely-timed, adaptive stimulation can suppress seizure transitions, by leveraging network silence as an early warning our work extends this paradigm and suggests new opportunities for targeted neuromodulation strategies to improve seizure control in epilepsy.

## Supplementary Information

Supplementary Information 1.

Supplementary Information 2.

Supplementary Information 3.

## Data Availability

The codes generated and analyzed during the current study are available in the GitHub repository (https://github.com/DiogoLeonai/Neuronal-silence-as-an-epileptic-seizure-biomarker). The datasets analyzed in this study were originally collected and published by Elahian et al.^40^ and are publicly available in the Zenodo repository^48^ (https://doi.org/10.5281/zenodo.836286).
