# Supplementary material for: Neuronal silence as a predictive biomarker and target for epileptic seizures suppression
Source: Sci Rep. 2026 Apr 9;16:16732. doi: 10.1038/s41598-026-44063-w (PMC13223270; doi:10.1038/s41598-026-44063-w)
Supplement: Supplementary file 1 — Supplementary Information 1. [file 41598_2026_44063_MOESM1_ESM.pdf]

Supplementary Information

Ion channels description

Traub and Miles [1] adapted the potassium and sodium currents, introduced by Hodgkin and Huxley, to better describe the electrical potential of central neurons. The adapted potassium current is given by

$$
\begin{aligned}
I^i_{\mathrm{K}} &= g_{\mathrm{K}} n_i^4 (V_i - E_{\mathrm{K}}), && (1)\\
\frac{dn_i}{dt} &= \alpha_{n,i}(V_i)(1-n_i) - \beta_{n,i}(V_i) n_i, && (2)\\
\alpha_{n,i}(V_i) &= \frac{-0.032(V_i - V_{\mathrm{T}} - 15)}{\exp\left(-\frac{V_i - V_{\mathrm{T}} - 15}{5}\right) - 1}, && (3)\\
\beta_{n,i}(V_i) &= 0.5 \exp\left(-\frac{V_i - V_{\mathrm{T}} - 10}{40}\right), && (4)
\end{aligned}
$$

where $g_{\mathrm{K}} = 5$ mS/cm^2 is the maximum conductance of the potassium channels, $E_{\mathrm{K}} = -100$ mV is the reverse potential of the potassium ions, n is the activation variable and $V_{\mathrm{T}} = -55$ mV is an adjustment of the peak threshold. The central neurons' sodium current is described by the following set of equations

$$
\begin{aligned}
I^i_{\mathrm{Na}} &= g_{\mathrm{Na}} m_i^3 h_i (V_i - E_{\mathrm{Na}}), && (5)\\
\frac{dm_i}{dt} &= \alpha_{m,i}(1-m_i) - \beta_{m,i} m_i, && (6)\\
\frac{dh_i}{dt} &= \alpha_{h,i}(1-h_i) - \beta_{h,i} h_i, && (7)\\
\alpha_{m,i} &= \frac{-0.32(V_i - V_{\mathrm{T}} - 13)}{\exp\left(-\frac{V_i - V_{\mathrm{T}} - 13}{4}\right) - 1}, && (8)\\
\beta_{m,i} &= \frac{0.28(V_i - V_{\mathrm{T}} - 40)}{\exp\left(\frac{V_i - V_{\mathrm{T}} - 40}{5}\right) - 1}, && (9)\\
\alpha_{h,i} &= 0.128 \exp\left(-\frac{V_i - V_{\mathrm{T}} - 17}{18}\right), && (10)\\
\beta_{h,i} &= \frac{4}{1 + \exp\left(-\frac{V_i - V_{\mathrm{T}} - 40}{5}\right)}, && (11)
\end{aligned}
$$

where $g_{\mathrm{Na}} = 50$ mS/cm^2 is the conductance of sodium channels, $E_{\mathrm{Na}} = 50$ mV is the reverse potential of sodium ions, m and h are the activation and inactivation channels variables, respectively.

The I^i_{Na} and I^i_{K} represent the fast dynamics of the sodium and potassium currents, respec-

tively. However, these descriptions lack some crucial mechanisms of ionic channels, e.g. adaptation [2, 3]. To incorporate adaptation, we include the slow potassium current I_{M}, originally described by Yamada et al. [2], which plays a key role in regulating neuronal adaptability. The expression for I_{M}^i current is given by

$$I_{\mathrm{M}}^i = g_{\mathrm{M}} p_i (V_i - E_{\mathrm{K}}), \tag{12}$$

$$\frac{dp_i}{dt} = \frac{p_\infty(V_i) - p_i}{\tau_p(V_i)}, \tag{13}$$

$$p_\infty = \frac{1}{1 + \exp\left(-\frac{V_i+35}{10}\right)}, \tag{14}$$

$$\tau_p(V_i) = \frac{\tau_{\max} \exp\left(-\frac{V_i+35}{20}\right)}{3.3 + \exp\left(-2\frac{(V_i+35)}{20}\right)}, \tag{15}$$

where $g_{\mathrm{M}} = 0.03$ mS/cm^2 is the conductance of the slow potassium channels and $\tau_{\max} =$ 1000 ms [3].

Random Forest algorithm

The Random Forest (RF) algorithm is a supervised machine learning method that belongs to the class of ensemble learning techniques. It consists of an ensemble of decision trees, where each tree is trained on a randomly selected subset of the training data (both in samples and features). For regression tasks, the final prediction is obtained by averaging the predictions of all individual trees, which reduces variance and improves generalization. This approach is particularly advantageous when modeling nonlinear and complex relationships, as it is less prone to overfitting compared to a single decision tree.

In this work, we applied the Random Forest regressor to predict the Kuramoto order parameter, $\mathrm{R}_{\mathrm{pred}}(\mathrm{t})$, at a given time t, using the mean silence time $\langle T \rangle$ at two previous time points, $t - \mathrm{lag}$ and $t - 2 \times \mathrm{lag}$, with $\mathrm{lag} = 10\,\mathrm{ms}$:

$$\mathrm{R}_{\mathrm{pred}}(t) = f\big(\langle T \rangle(t - \mathrm{lag}), \langle T \rangle(t - 2 \times \mathrm{lag})\big), \tag{16}$$

where $\langle T \rangle(t - \mathrm{lag})$ is the delayed mean silence time. Figure S1 illustrates the general workflow of the Random Forest regression procedure.

We implemented the RF model using the `scikit-learn` Python library. The dataset corresponding to the coupling strength $g_{\mathrm{syn}} = 1.811\,\mu$S/cm^2 was split evenly into training (50%) and testing (50%) sets. While this dataset was used for model training, silence time series from other coupling strengths could also serve as training data. Hyperparameters were optimized via a grid search to enhance predictive performance. The selected

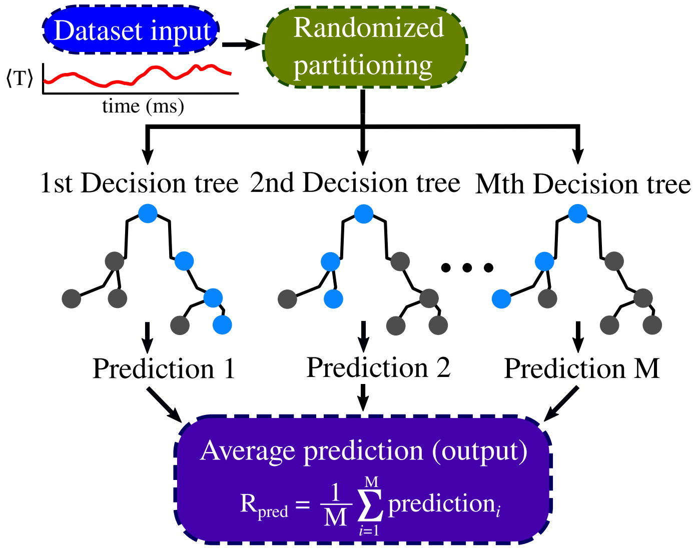

Figure S1: Illustration of the Random Forest algorithm. The Random Forest is composed of multiple decision trees, each trained on a random subset of the training data. The final prediction is made by averaging the predictions of all trees.

configuration was: `criterion` = `absolute_error`, `n_estimators` = 200, `max_features` = 0.2, `max_depth` = 30, and `random_state` = 18.

To evaluate generalization, we trained the model exclusively on the $g_{syn} = 1.811\,\mu S/cm^2$ $\langle T \rangle$ dataset and then predicted R_{pred} for mean silence time of different coupling strengths. Figure S2 displays the simulated $R(t)$ (in red) and the R_{pred} (in black) for two different couplings, the RF was trained only with 50% of the $g_{syn} = 1.811\,\mu S/cm^2$ dataset. Panel (a) displays the results for $g_{syn} = 1.825\,\mu S/cm^2$, panel (b) shows the results for $g_{syn} = 1.830\,\mu S/cm^2$, and panel (c) depicts the results for $g_{syn} = 1.835\,\mu S/cm^2$. The model successfully predicts the Kuramoto order parameter, demonstrating its ability to generalize across different coupling strengths.

Raster plots

Figure S3 shows the raster plots (top panels) and the mean silence time (bottom panels) for four different time windows with $g_{syn} = 1.835\,\mu S/cm^2$: (a) $t = [46, 56]$ s, (b) $t = [148, 160]$ s, (c) $t = [195, 204]$ s, and (d) $t = [210, 220]$ s. Across these time windows, desynchronization is evident in the raster plots, accompanied by a low mean silence time during the down states. In some mean silence time plots, small peaks can be observed preceding the rapid increase of the mean silence, however, these features do not consistently appear before every down-to-up transition.

Figure S4 displays raster plots of an up state for $g_{syn} = 1.835$ under different suppression conditions. Panel (a) shows the up state without any suppression, while panels (b–

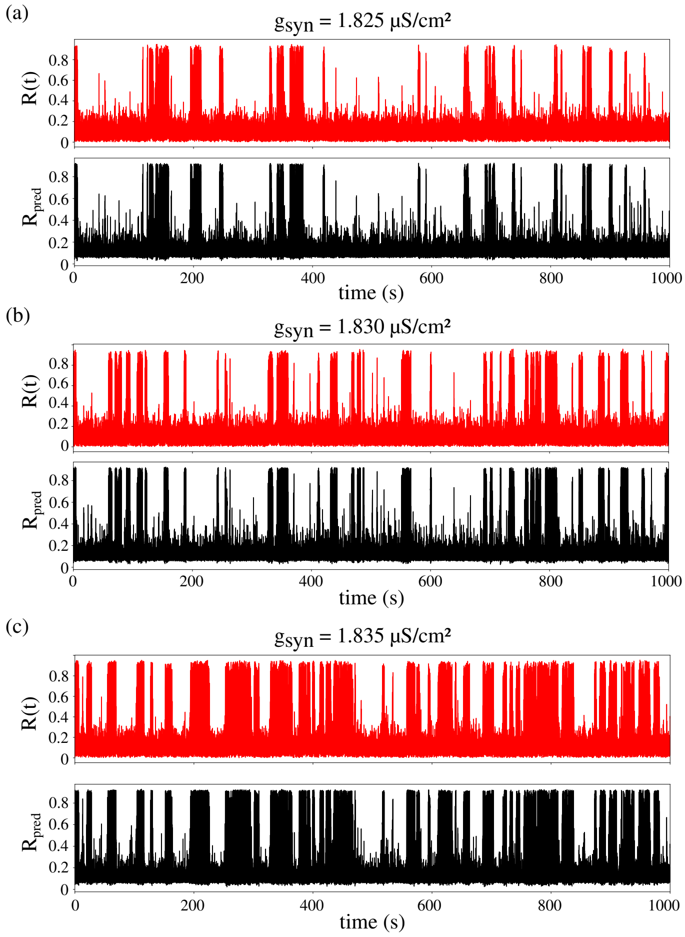

Figure S2: Random Forest prediction of the Kuramoto order parameter for different coupling strengths. The red line represents the simulated Kuramoto order parameter R(t), while the black line shows the predicted order parameter $\mathrm{R}_{\mathrm{pred}}(t)$ using the Random Forest algorithm. Panel (a) corresponds to $g_{\mathrm{syn}} = 1.825\,\mu\mathrm{S/cm}^2$, panel (b) corresponds to $g_{\mathrm{syn}} = 1.83\,\mu\mathrm{S/cm}^2$, and panel (c) corresponds to $g_{\mathrm{syn}} = 1.835\,\mu\mathrm{S/cm}^2$. The model was trained only with 50% of the data of $g_{\mathrm{syn}} = 1.811\,\mu\mathrm{S/cm}^2$.

e) illustrate the effects of varying suppression currents (I_{sup}) and durations (t_{sup}): (b) $I_{\mathrm{sup}} = 40$ pA, $t_{\mathrm{sup}} = 0.5$ s; (c) $I_{\mathrm{sup}} = 200$ pA, $t_{\mathrm{sup}} = 0.5$ s; (d) $I_{\mathrm{sup}} = 40$ pA, $t_{\mathrm{sup}} = 1$ s; (e) $I_{\mathrm{sup}} = 200$ pA, $t_{\mathrm{sup}} = 1$ s. As shown in panel (b), this relatively weak and brief suppression current is insufficient to reduce the up-state duration from the approximately 6 s observed in panel (a). Increasing the duration of the same current (panel d) shortens the up-state duration, whereas a stronger suppression current ($I_{\mathrm{sup}} = 200$ pA; panels c and e) effectively reduces the up-state duration even with a brief application.'

Video V1 depicts the down-to-up state transition in the cortical network. Video V2 compares the network dynamics in the absence and presence of the suppression current, I_{sup}. In the simulation, the upper panels show the case without suppression current, while the lower panels display the case with suppression current applied.

Video V1. Neuronal activity of the cortical network in the down-to-up state transition.

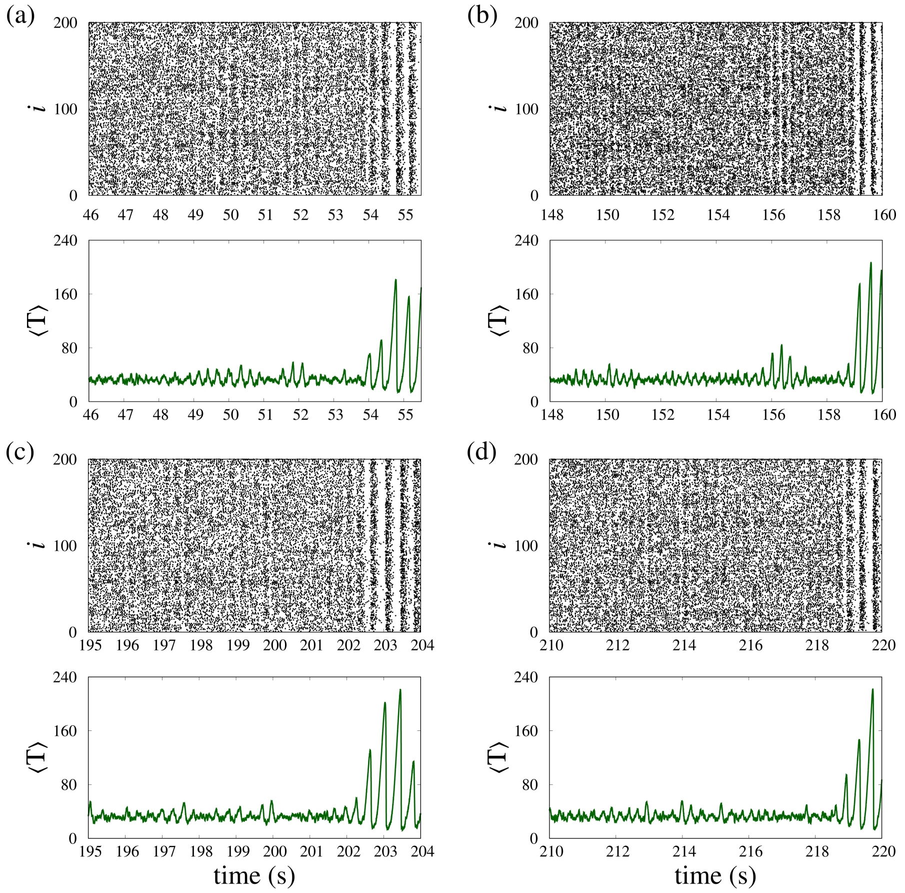

Figure S3: Rasters and mean silence time shown in the top and bottom panels, respectively, for $g_{\mathrm{syn}} = 1.835\,\mu\mathrm{S/cm}^2$, (a) time= $[46, 56]$ s, (b) time= $[148, 160]$ s, (c) time= $[195, 204]$ s, and (d) time= $[210, 220]$ s.

Video V2. Comparison of network activity in absence vs. presence of suppression current I_{sup}.

References

[1] R. D. Traub and R. Miles. *Neuronal networks of the hippocampus*. Cambridge University Press, Cambridge, 1991.

[2] W. M. Yamada, C. Koch, and P. R. Adams. Multiple channels and calcium dynamics. In C. Koch and I. Segev, editors, *Methods in Neuronal Modeling: From Synapses to Networks*, pages 97–134. MIT Press, 1989.

[3] M. Pospischil, M. Toledo-Rodriguez, C. Monier, Z. Piwkowska, T. Bal, Y. Frégnac, and A. Destexhe. Minimal hodgkin–huxley type models for different classes of cortical and thalamic neurons. *Biological Cybernetics*, 99(4-5):427–441, 2008.

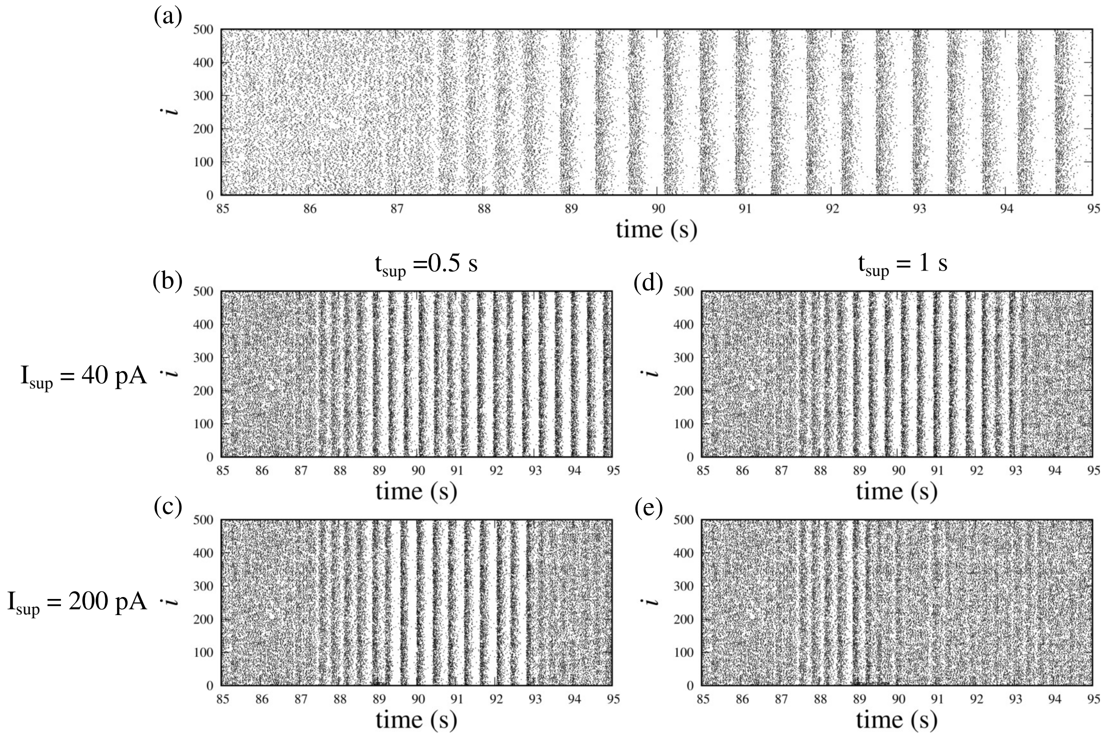

Figure S4: Raster plots of an up state under different suppression currents (I_{sup}) and durations (t_{sup}). (a) Up state for $g_{\text{syn}} = 1.835$ without suppression. Panels (b–e) show the same up state under varying suppression conditions: (b) $I_{\text{sup}} = 40$ pA, $t_{\text{sup}} = 0.5$ s; (c) $I_{\text{sup}} = 200$ pA, $t_{\text{sup}} = 0.5$ s; (d) $I_{\text{sup}} = 40$ pA, $t_{\text{sup}} = 1$ s; (e) $I_{\text{sup}} = 200$ pA, $t_{\text{sup}} = 1$ s.
